# Complete Diphallia With Associated Epispadias: A Rare Congenital Anomaly Diagnosed in Adulthood

**DOI:** 10.7759/cureus.103891

**Published:** 2026-02-19

**Authors:** Shuvo Khandokar, Sabrin Islam

**Affiliations:** 1 Surgery, Shaheed Ziaur Rahman Medical College, Bogura, BGD

**Keywords:** anorectal malformation, congenital anomaly, diphallia, penile duplication, urogenital anomaly

## Abstract

Diphalia is an extremely rare urogenital condition presenting with penile duplication. Although it is typically diagnosed in the neonatal period due to associated congenital anomalies, some cases may present late without any related complications. Every case of diphallia presents a distinct set of symptoms and associations. In this case report, we underscore an interesting clinical presentation of diphallia in a 24-year-old male and the significance of individualized management of the condition.

## Introduction

Diphallia, also known as penile duplication, is a rare congenital malformation that occurs in 1 in 5 million human births. The first reported case dates back to 1609 by Johannes Jacob Wecker in Bologna [1]. The anomaly ranges from partial to complete duplication of the penis. Each case has a unique presentation. Diphallia has been reported in association with duplication of the bladder, urethra, and colon, as well as anorectal malformations and vertebral anomalies [2].

Most reported cases are diagnosed in the neonatal period due to associated congenital abnormalities and functional complications, particularly involving the genitourinary and gastrointestinal systems. However, some cases remain undetected until adulthood, when patients present with urinary, reproductive, or psychosocial concerns. Due to the rarity of the condition and the wide phenotypic spectrum, universal management guidelines do not exist [3].

Management is individualized and depends on associated anomalies, urinary continence, erectile function, and cosmetic considerations. While surgical reconstruction is often performed in childhood for functional impairment, conservative management may be appropriate in asymptomatic patients with preserved function [3,4].

We present a rare case of diphallia diagnosed incidentally in adulthood without associated malformations and managed conservatively.

## Case presentation

A 24-year-old male was admitted to the surgery inpatient department with complaints of recurrent right upper abdominal colicky pain associated with nausea and vomiting. Thorough general and systemic examination, abdominal ultrasonography, and other routine examinations revealed the case to be chronic calculus cholecystitis.

Upon exposure to abdominal examination, we incidentally found another finding unrelated to the presenting symptoms. The patient possessed two phalli. It had been present since his birth. As per religious rituals, circumcision was performed on both phalli as well. Apart from urinary spraying and difficulty in directing the stream, he did not complain of any inconvenience.

**Figure 1:**
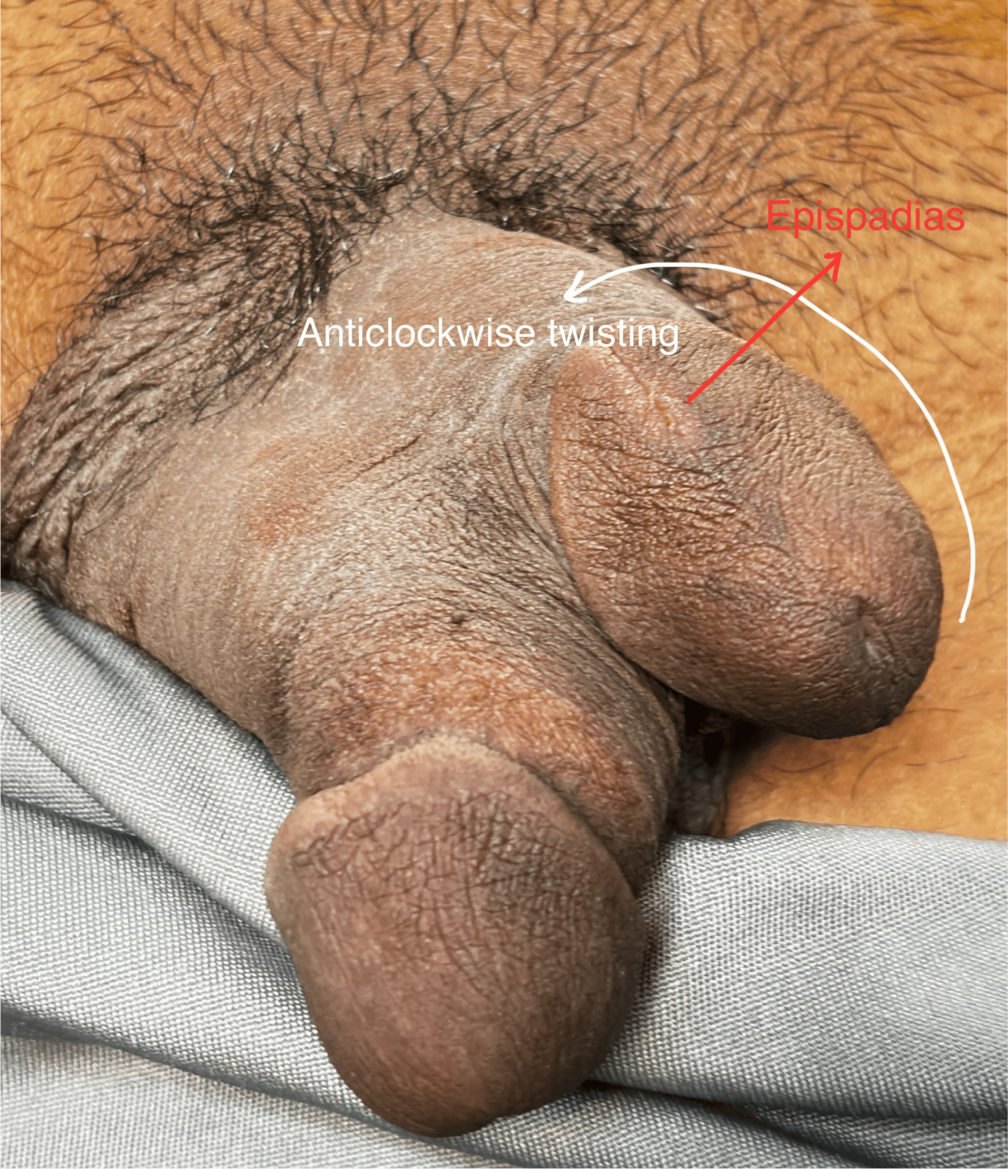
View from the front: axial twisting and epispadias is notable.

**Figure 2:**
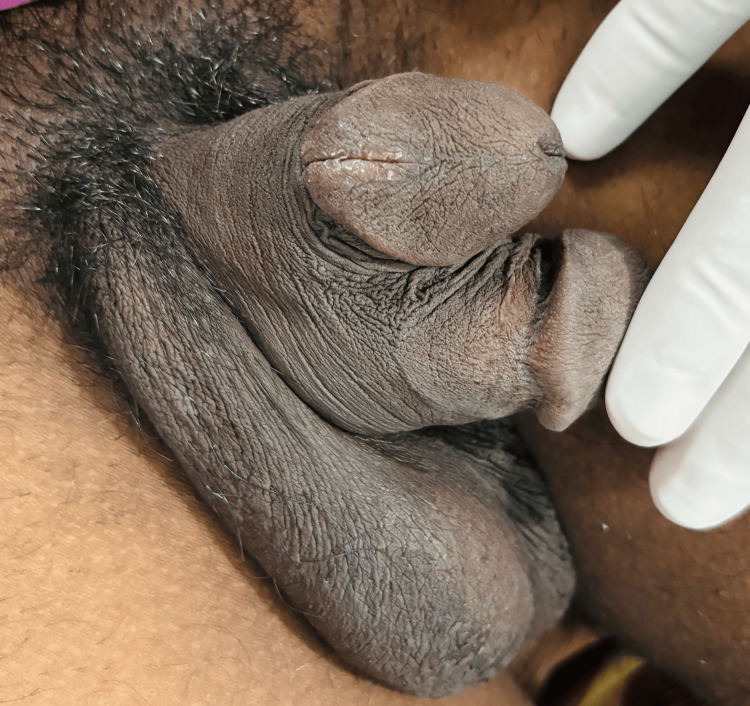
Lateral view from the right side.

**Figure 3:**
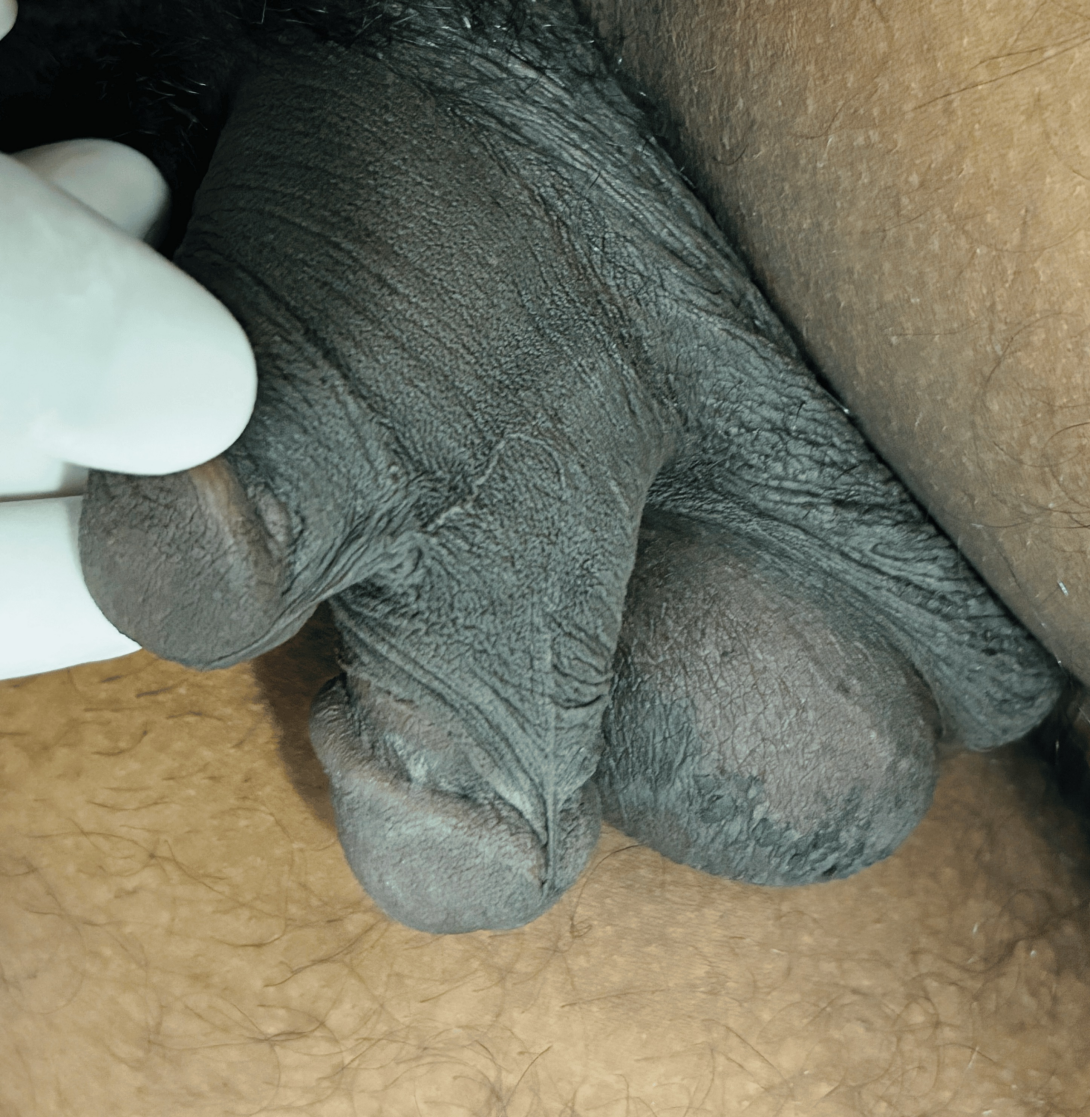
Left lateral view showing penile raphe on the left lateral side denoting anticlockwise twisting of the shafts.

Physical examination of the pubic area revealed two distinct penises arising from a common pubic base. Both penises were well developed, one stacked above another, each of which contained two palpable corpora cavernosa. While both of the phalli had functional urethral meatus, the ventral shaft was dominant in size, having a single urethral meatus and most urinary flow, which made it the most normal of the two shafts. The dorsal shaft contained two urethral meatus: one at the tip of the glans and another presenting as epispadias near the corona glandis (Figure 1). Considering these characters, the diphallia can be categorized as type 1Aα as per the proposed surgical classification system by Kendrick and Kimble [1]. The shafts, along with all three urethral meatus were rotated anticlockwise along the penile axis (Figures 1-3). The scrotum showed normal anatomy with bilateral descended testes of normal size and consistency. The patient did not complain of erectile dysfunction, dysuria, hematuria, urinary tract infections, or hematuria.

Laboratory investigations, including ultrasonography of the whole abdomen, complete blood count, ECG, serum creatinine, liver function tests, random blood sugar, and chest X-ray, revealed no abnormality. Sonographical findings included a single urinary bladder, bilateral ureter, and kidneys with normal configuration. No associated anorectal, vertebral, or other systemic anomalies were identified.

After consultation with the urology department, the patient was informed about possible interference in personal lifestyle and counseled for further management of the condition. Given preserved urinary function and absence of any other disturbance other than inconvenience in urination at present, the patient preferred a conservative approach with follow-up.

## Discussion

Diphallia is an exceptionally rare anomaly. The embryologic basis of the condition is not fully understood. Two proposed mechanisms are separation of the pubic tubercles, in which each phallus contains one corporal body and one urethra; or cleavage of the pubic tubercle, whereby each phallus has one complete pair of corporal bodies and urethras [5].

Most reported cases are associated with urogenital anomalies and/or anorectal malformations. Associated urogenital anomalies include hypospadias and epispadias in either or both the phalli, exstrophy bladder, duplication of bladder, and scrotal duplications [6,7]. Recently published data support the hypothesis that early mesodermal disruption can simultaneously affect the genital tubercle, urinary tract, and axial skeleton [8].

Additional reports in the literature emphasize that the clinical spectrum of diphallia varies widely, ranging from isolated duplication of the glans to complete penile duplication with complex multisystem malformations. Imaging modalities such as ultrasonography, voiding cystourethrography, and MRI play an important role in delineating urethral anatomy and identifying associated anomalies before planning intervention [4]. Careful anatomical assessment is essential for selecting an appropriate management strategy.

Several case reports and reviews highlight that preservation of urinary continence and erectile function remains the primary goal of treatment. Surgical intervention is therefore tailored to individual anatomy and symptom burden rather than the duplication itself. In patients without obstruction, infection, incontinence, or significant cosmetic concern, observation with periodic follow-up has been reported as a safe approach [9].

Adult presentation is uncommon and may be attributed to social stigma, limited access to healthcare, or preserved functional status during childhood. In adults, management focuses not only on cosmetic goals but also on urinary function, sexual performance, fertility, and psychological well-being. Long-term prognosis largely depends on the presence and severity of associated congenital anomalies rather than penile duplication alone. Psychosocial counseling and multidisciplinary evaluation involving urology, radiology, and mental health professionals may be beneficial, particularly in patients presenting later in life [4,9].

## Conclusions

Complete diphallia is a rare congenital anomaly that may occasionally present in adulthood. Meticulous evaluation for associated anomalies, even in clinically stable or minimally symptomatic patients, along with careful consideration of patient preferences, is critical for determining the appropriate management. Individualized treatment plans and long-term follow-up are essential to optimize functional and psychosocial outcomes.
